# Patient Perspectives on the Challenges and Responsibilities of Living With Chronic Inflammatory Diseases: Qualitative Study

**DOI:** 10.2196/10815

**Published:** 2018-11-21

**Authors:** Graham George Macdonald, Cheryl Koehn, Gail Attara, Allan Stordy, Marilee Allerdings, Jenny Leese, Linda C Li, Catherine L Backman

**Affiliations:** 1 Arthritis Research Canada Richmond, BC Canada; 2 Rehabilitation Sciences Graduate Program University of British Columbia Vancouver, BC Canada; 3 Arthritis Consumer Experts Vancouver, BC Canada; 4 Canadian Society of Intestinal Research Vancouver, BC Canada; 5 Canadian Skin Patient Alliance Vancouver, BC Canada; 6 Arthritis Patient Advisory Board Arthritis Research Canada Vancouver, BC Canada; 7 Department of Physical Therapy University of British Columbia Vancouver, BC Canada; 8 Department of Occupational Science and Occupational Therapy University of British Columbia Vancouver, BC Canada

**Keywords:** patient participation, chronic disease, arthritis, inflammation

## Abstract

**Background:**

Collectively, chronic inflammatory diseases take a great toll on individuals and society in terms of participation restrictions, quality of life, and economic costs. Although prior qualitative studies have reported patients’ experiences and challenges living with specific diseases, few have compared the consequences of disease management in daily life across different types of inflammatory diseases in studies led by patient partners.

**Objective:**

The aim of this study was to identify the significant consequences of inflammatory arthritis, psoriasis, and inflammatory bowel diseases on daily life and explore commonalities across diseases.

**Methods:**

A cross-sectional Web-based survey was designed by patient research partners and distributed by patient awareness organizations via their social media channels and by sharing a link in a newspaper story. One open-ended item asked about burdens and responsibilities experienced in daily life. Informed by narrative traditions in qualitative health research, we applied a thematic content analysis to participants’ written accounts in response to this item. This is an example of a study conceived, conducted, and interpreted with patients as research partners.

**Results:**

A total of 636 Canadians, with a median age band of 55-64 years, submitted surveys, and 80% of the respondents were women. Moreover, 540 participants provided written substantive responses to the open-ended item. Overall, 4 main narratives were generated: (1) daily life disrupted; (2) socioeconomic vulnerabilities; (3) stresses around visible, invisible, and hiding disabilities; and (4) actions aimed at staying positive. Ways in which participants experienced social stigma, pain and fatigue, balancing responsibilities, and worries about the future appeared throughout all 4 narratives.

**Conclusions:**

People living with chronic inflammatory diseases affecting joints, skin, and the digestive tract report important gaps between health, social, and economic support systems that create barriers to finding the services they need to sustain their health. Regardless of diagnosis, they report similar experiences navigating the consequences of lifelong conditions, which have implications for policy makers. There is a need for outcome measures in research and service delivery to address patient priorities and for programs to fill gaps created by the artificial administrative separation of health services, social services, and income assistance.

## Introduction

### Background

Patient engagement in health research has been building over the last two decades, with examples of effective collaborations between patients and researchers being reported with increasing frequency. The benefits of patient engagement across the research process include identifying research questions of greater relevance to patients’ concerns, improved participant enrollment and retention rates, and knowledge translation strategies that are more readily understood or adopted by community members [1]. Benefits to patients involved as investigators or research partners include a sense of empowerment, confidence, and contribution to the greater good that arises from meaningful engagement in the research process from inception to dissemination [2]. This paper describes findings from a project led by patient research partners. It describes the consequences of inflammatory arthritis, psoriasis, and inflammatory bowel diseases on daily life and explores commonalities across diseases.

In particular, this paper focuses on inflammatory types of arthritis (such as rheumatoid arthritis, ankylosing spondylitis, and psoriatic arthritis), psoriasis, Crohn disease, and ulcerative colitis. All of these are systemic, autoimmune conditions [3-5]. Their clinical presentation ranges from mild to severe, and they are characterized as episodic, meaning people live with the uncertainty of exacerbations and remissions either from the natural course of the disease or its medical management [3-5]. People who have 1 disease, for example, psoriasis, are at higher risk of concurrently having one of the other diseases, for example, arthritis or Crohn disease [4]. Studies on the impact of living with these inflammatory conditions show disruption to normal daily activities [6-10], reduced productivity [9-11], and high personal costs because of the loss of ability to work and medical and other costs associated with health maintenance, which threaten financial security [6,11].

Among women with early rheumatoid arthritis, McDonald et al found that the uncertainty of having an episodic illness with fluctuating symptoms was particularly problematic as women experienced good days (able to engage in typical routines and daily activities), bad days (experiencing limitations in typical routines and daily activities), and worse days (often halting usual activities because of pain, fatigue, or recovering from symptom flares) [12]. Adapting to activity disruption threatened self-identity and sense of self [13]. Similar experiences have been reported by adults living with established inflammatory bowel disease [14,15] and psoriasis [13,16,17]. For example, among men and women with inflammatory bowel disease, unpredictable symptoms restricted social activities, employment, travel, and shopping, presenting enormous challenges to leading a normal life or maintaining the appearance of normality to others [14]. A survey of Canadians with Crohn disease or ulcerative colitis reported participation restrictions in leisure activities and interpersonal relationships to be the most frequently reported consequences of the disease, at 64% and 52%, respectively [6].

### Objectives

Given the frequency of activity disruptions reported in these (and other) qualitative studies, which by nature focus on relatively small numbers of participants, we recognized an opportunity to draw connections across disease groups with a larger number of participants. Such studies are valuable to patients because they corroborate their experiences; show they are not alone; and provide strategies for living well, interacting with health professionals, and advocating for resources. They are valuable to professionals for enhanced understanding of the impact of living with different diseases, placing patient experiences in context, and ultimately help improve patient-provider communication for more compassionate care [18]. By inviting a large number of people to respond to an open-ended question typical of qualitative research, this study potentially verifies and extends the transferability of findings from small studies. The study examines similarities and differences across respondents with a wide range of inflammatory diseases. Its specific purpose is to describe the consequences of inflammatory arthritis, psoriasis, and inflammatory bowel diseases on daily life and explore commonalities across diseases.

## Methods

### Design

A cross-sectional descriptive design was used with a Web-based survey. This paper focuses on written text responses using qualitative content and narrative analysis. Ethical approval was obtained from the behavioral ethics review board of the researchers’ university.

### Study Context and Role of Patient Partners

It has been recommended that patient and public involvement in research be explicitly reported [19]. Each of the 4 patient research partners (CK, AS, GA, and MA) is affiliated with a national public awareness or charitable organization focused on education, information sharing, and encouraging research. Two are members of organizations focused on arthritis and joint diseases, 1 works with an organization for gastrointestinal and inflammatory bowel diseases, and 1 with an organization for psoriasis and inflammatory skin diseases. They volunteered as consumer and patient partners along with researchers to develop a grant application in response to a specific call for proposals to fund research teams with a focus on chronic inflammatory diseases. The bid was successful, creating PRECISION, a pan-Canadian team of over 30 researchers including patients working on a series of interconnected studies.

PRECISION is an acronym for PREventing Complications from Inflammatory Skin, joint, and bowel conditIONs, and the diseases under study are psoriasis, rheumatoid arthritis, lupus, ankylosing spondylitis, gout, Crohn disease, and ulcerative colitis. The team objectives include assessing the risk and burden of complications and consequences of these diseases and testing novel health services aimed at preventing or mediating those complications, priorities identified through patient-researcher collaboration [20]. This context is important because this study is a direct consequence of the way patients chose to inform PRECISION’s objectives.

The patient partners designed a survey to gather data to strengthen the patient perspective component of the grant application. When the volume and depth of data received was greater than anticipated, a systematic data analysis plan was developed in collaboration with 4 PRECISION researchers to give voice to the concerns raised by survey respondents. The role of the patient partners in this paper thus included survey design and implementation, participant recruitment, assistance throughout data analysis and interpretation, and review of manuscript drafts.

### Participants

The patient partners, through the social media and e-newsletters of their 4 organizations, distributed the survey link to their subscribers nationwide. The patient partners also connected with a newspaper reporter who wrote a brief story that included the survey link in the print version of a metropolitan daily newspaper and the reporter’s blog. There were no explicit inclusion criteria other than the survey notice that specifically invited people with inflammatory joint, skin, or bowel diseases to have a say in research and complete the survey anonymously. Consent was implied by submitting a completed survey. The survey was open for 3 weeks in the summer of 2013 and was hosted online on SurveyMonkey.

### Survey Content

Patient research partners designed a Web-based survey to identify patient priorities for research to help justify the objectives of PRECISION. The patient research partners invited all team members to contribute items for inclusion in the survey and then vetted a large number of potential items to reduce the total number and ensure clarity of the retained items. In addition to basic demographic information (eg, diagnosis, sex, age group in 10-year age bands, and urban vs rural place of residence), the survey contained closed-response and open-ended items to gather patient perspectives on medication use, knowledge about potential disease complications, treatments and interventions, lifestyle habits (eg, physical activity), and experiences living with inflammatory diseases. The responses to closed-response items helped justify the grant application with respect to needs around specific diseases, complications, medications, and physical activity [21].

In this paper, we focus on text responses to the following open-ended question: *what are some of the burdens and responsibilities you face in managing or living well with your illness?* There was no word limit imposed on stories written in response to this item nor was it required that participants enter any text.

### Data Analysis

Responses were downloaded verbatim into an Excel file for tabulation (keeping text responses linked to demographic descriptors such as age and diagnosis) and analysis. The burdens and responsibility question generated numerous stories and commentary. Tallying was avoided because the spontaneous responses to the open-ended question meant that some respondents introduced new topics that, if tallied, would not represent the proportion of respondents who shared that view; counting was not found to yield specific or meaningful data [22]. Accordingly, we drew upon narrative traditions that allow personal accounts and experiences to conduct a thematic content analysis of these text responses [23,24]. We sought to understand *what* people experienced rather than *how* they described it, making thematic content analysis more appropriate than other forms of narrative models for this dataset [23]. Thematic content analysis is suitable to participatory types of research because it is generally understandable by all audiences, highlights similarities and differences within the dataset, and allows for socially relevant interpretations to inform policy development [25].

Trustworthiness depends in part on the description of the analytical process. We read and reread all responses to become familiar with the data and then identified common and repeated elements to broadly classify the issues and topics of concern to respondents. Our analysis began with open coding of the data in which we flagged phrases of interest from the responses. The initial codes were then clustered into categories based on recurring elements and common subjects. These categories were then analyzed for the character of the responses they contained and their narrative context. Categories were further clustered to derive tentative themes. Themes were then discussed and agreed upon by the team through discussion and review of written descriptions with supporting quotes. The final analysis was represented by 4 narrative themes.

### Validating the Analysis

The preliminary content analysis was developed by 2 researchers (GGM and CLB) with qualitative research experience, who brought different lenses to the dataset (one is male, early career, and educated in the social sciences; the other is female, health professional, and senior researcher). The preliminary topics and supporting evidence (data extractions) were discussed by all coauthors at a team meeting, and draft categories were developed and circulated by email, and additional comments and interpretations were gathered through sequential iterations appraising data and interpretations. As the patient partners were representatives of organizations each dedicated to different disease groups, their feedback served as a form of member checking as to whether findings resonated with experiences and concerns of their respective groups.

The 4 patient partners and 4 researchers thus co-constructed narratives reflecting the common experiences within the dataset and agreed upon quotes to represent each narrative. Collectively, the 8 collaborators bring perspectives from men and women, young adult to late middle-aged, and health care, research, or lived experience across inflammatory skin, joint, and bowel diseases, experiences that contribute to the trustworthiness of interpretations. As a final step to enhance transparency and trustworthiness, the analytical process and findings were reviewed with a peer experienced in qualitative methodology and health research.

## Results

### Demographics

We received 636 unique surveys. Respondents’ age varied from 18-24 years to 85-94 years (median age band 55-64 years), and 80.0% (509/636) of the respondents were women, which reflects the higher prevalence of women affected by most of the diseases in this study. The majority, 71.1% (452/636), were from British Columbia (the location of the newspaper with the survey link), with additional respondents from all other Canadian provinces and 2 territories. Most (91%) lived in a city with at least one hospital. Moreover, 42.9% (273/636) reported multiple health conditions, often 2 of the 3 inflammatory disease categories part of PRECISION, for example, Crohn disease and arthritis. Consequently, the following proportions sum beyond 100%: 86.0% (547/636) reported inflammatory joint diseases, 25.9% (165/636) reported psoriasis, and 18.1% (115/636) reported inflammatory bowel diseases.

Of the 636 respondents, 540 (85.0%) responded to the burdens and responsibilities question. These varied in length from a single phrase (eg, “maintaining mobility and managing pain when I have flare-ups”) to lengthy accounts of concerns for themselves and their families, descriptions of living with their disease or diseases in daily life, and efforts to take charge of their unique situation. Overall, responses outlined the ways in which the health care system and society in general are both helping and failing this population.

Overall, 4 key narratives were crafted to represent the substance of the large number of text entries: daily life disrupted; visible, invisible, and hidden disability; socioeconomic vulnerability; and staying positive. Verbatim data show considerable overlap among the themes; therefore, some quotes easily support more than 1 key narrative. Examples of social stigma, pain and fatigue, balancing work and family responsibilities, and worries about the future contributed to all 4 narratives. For example, *experiencing* symptoms such as pain and fatigue were precursor to the first 3 narratives related to disruptions in daily life, disability perceptions, and social vulnerabilities, and *coping* with symptoms was apparent in staying positive, the fourth narrative. Each narrative is described below; alphanumeric labels link to quotations in Tables 1-3. Each quote references the sex, age band, province or territory of residence (using postal abbreviation), and reported diagnosis.

### Daily Life Disrupted

Respondents told stories characterized by disruptions to tasks, activities, and roles, ranging from inconveniences to major shifts in how they participated in life. The most frequently cited antecedent to disrupted activities was persistent and sometimes unrelenting pain and fatigue, reported by more than half of the sample. Managing symptoms necessitated setting priorities that tended to place obligatory work or household responsibilities ahead of equally important but more discretionary activities such as maintaining social connections or enjoyable leisure activities (Table 1: A1 and A2). Although employment was often stated as high priority, many respondents struggled to sustain participation in work. Repeatedly, respondents outlined difficulties fulfilling the roles that others expected of them or shared serious concerns for the future if they were unable to continue work or take care of their own health (Table 1: A3 and A4).

Descriptions of disrupted daily routines and the need for planning ahead were more often reported by those with joint or bowel diseases than those with skin conditions. Disruption was a prominent narrative in social situations, and some found it very stigmatizing to “say no to social activities” and “curtail my hobbies and be vigilant of travel plans” to manage symptoms. Respondents experienced adversity in their social environments, feeling forced to adapt to circumstances and relationships that did not give credence to their illness experience (Table 1: A2). They reported concerns about being inadequate as friends, partners, or family members, and some expressed feeling inferior to their peers at work. Respondents with inflammatory bowel diseases reported constant stress over whether or not they will be able to access a bathroom facility at a moment’s notice as curtailing social interaction (Table 1: B1 and B2).

### Visible, Invisible, and Hiding Disability

Collectively, descriptions debated the extent to which these conditions are or are not visible, how that affects interpersonal relationships, and whether or not there is a need to consciously hide disability. A clear cluster of responses related to appearing sick versus well, of how “looking well does not always mean feeling well” and how this could be burdensome when trying to “give your family a break from your disease. Relationships take a beating.” Visible disease characteristics such as psoriatic plaques affected relationships (Table 2: C1 and C2).

Although some respondents spoke about visible characteristics of their diseases, there were more descriptions of how invisible disability (appearing normal) led to individuals feeling marginalized (Table 2: D1) and wanting to explain, increase awareness, or find a way to foster understanding, assistance, or universal accessibility (Table 2: D2). Family, social, and employment relationships were reported to suffer because of a lack of empathy and understanding:

I guess the biggest casualty is that I never had the energy to create an active social life. A lot of people do not understand last minute cancellations for plans because all of a sudden you lack the energy to participate.

Female, BC, 55-64 years, psoriatic arthritis

At times, respondents described feeling devalued by society as weak or dysfunctional and how those attitudes are held or contested by their spouses, friends, coworkers, or bosses (Table 2: D3 and D4). In contrast, other descriptions related to the desire or perceived need to hide the disease. Sometimes, this reflected wanting to participate in activities like anyone else, whereas other examples related to fears about being treated differently or losing opportunities or jobs (Table 2: E1 and E2).

**Table 1:** Daily life disrupted.

Subtheme	Quotes
Accommodating symptoms disrupts work	A1: *The largest burden is the effect fatigue has on social and work life*- *I have had to adapt my sleep patterns so I can perform at work; in the end my social life suffers.* [Female; 25-34 years; SK^a^; RA^b^]
	A2: *The disease is sometimes invisible and people don't understand issues with fatigue or sudden onset of pain/flares. This has a profound impact in the work place and in personal relationships. I am often perceived as lazy when I can't get mobile in the morning (late for work) and tend to over-compensate by working late and taking on more than I can manage. This pattern will lead to a flare which continues in a downward cycle. I hesitate to ask for help because I don't look sick (don't use a walker or cane) and am often judged to be “weak.” In one workplace, a supervisor told my coworkers not to coddle me and that I had a low pain threshold. My partner has a hard time understanding the fatigue part but is very sympathetic to painful flares.* [Female; 45-54 years; BC^c^; psoriatic arthritis]
	A3: *I don't have the energy to do what I feel needs to be done, nor do I have the physical ability to do it. I want a pain free / tired free day. [I am] feeling inadequate in many aspects, losing my independence, always in pain, not being able to continue with my well-paying job.* [Female; 55-64 years; BC; RA and OA^d^)]
	A4: *Working full time and being a mom to 20 month old is very challenging, never mind finding time and energy to exercise, or even just be able to get enough rest.* [Female; 35-44 years; ON^e^; RA]
Disruptions specific to inflammatory bowel diseases	B1: *The thing of most concern is that the only treatments mostly involving taking drugs with very significant and unpleasant (and dangerous) side effects. I know many with UC [ulcerative colitis] feel a bit like guinea pigs as we try to control our disease and be active and contributing members of society. Ulcerative colitis is very unpredictable. I have tried many “alternative” things, none of which have helped. I have experienced flares dozens of times and I have no idea why they happen when they do. The need for a washroom quickly is a huge burden. Also, public washrooms are neither private nor soundproof. It causes me emotional strain to wait in a stall until everyone has left, only to have someone else come it. It is embarrassing and very few people understand. I cannot always participate in activities with family and friends. I don't think they understand. I often feel they think I am making excuses (which I do sometimes to get around saying it is my colitis). I worry about the impact of my disease on my work. I worry very much that it will make my retirement less than what I dream of. I feel like a burden to my husband sometimes. I feel I complain too much although I try not to. I cannot talk to anyone about my actual symptoms as they are considered disgusting.* [Female; 55-64 years; BC; UC^f^]
	B2: *Always looking to find a bathroom in case you need one, always having to explain to other people why you can't do or eat something, being disappointed in yourself because you can't do what everyone else is doing, disappointing your family (children) when you have to cancel plans at the last minute due to a flare up.* [Female; 65-74 years; BC; UC]

^a^SK: Saskatchewan.

^b^RA: rheumatoid arthritis.

^c^BC: British Columbia.

^d^OA: osteoarthritis.

^e^ON: Ontario.

^f^UC: ulcerative colitis.

**Table 2:** Visible, invisible, and hiding disability.

Subtheme	Quotes
Visible disability	C1: *Red raised patches and skin flakes all over my body interferes with my interpersonal relations and social life.* [Male; 55-64 years; AB^a^; psoriasis]
	C2: *Unsightliness of plaques. Annoyance as I have psoriasis. People’s comments and the unpleasant look of it.* [Female; 45-54 years; BC^b^; psoriasis, Sjogren syndrome]
Invisible disability	D1: *Having an invisible disease comes with a lot of judgmental bigots’ attention. Having accidents because of denied use of washrooms, being overweight because of the side effects of the medications. The pain can be crippling. Imagine not having ANY control over when and where you need to go to the washroom...while your body is in pain.* [Female; 25-34 years; BC; Crohn, RA^c^]
	D2: *[That] some people have a difficult time understanding or even believing I have to contend with illness can be trying. I was so surprised by what happened to me and by my diagnoses that I want to help others understand the complexities of rheumatic diseases.* [Female; 55-64 years; QC^d^; AS^e^, psoriasis, Crohn, Sjogren syndrome]
	D3: *Chronic pain and unpredictable flare-ups make it hard to manage life on a daily basis. I always have to carry painkillers with me. Often, I have trouble riding a bus or subway because I have limited mobility and joint strength. Navigating in public is hard when others do not seem to understand that someone who looks “normal” has trouble turning a doorknob, or holding a door open.* [Male; 55-64 years; BC; RA, psoriatic arthritis]
	D4: *Trying to manage a balance of work and family life while having chronic pain. Stress of not being able to support child if I have to take days off work. Being sick but not looking like a sick person is difficult as people don't understand.* [Female; 35-44 years; BC; lupus, Hashimoto disease]
Hiding disability	E1: *[It is a burden] making people aware of the disease without having a label put on you. Since my disease is not visible it is hard to hide pain.* [Female; 65-74 years; BC; AS]
	E2: *Being disciplined all the time. Not being a burden on my significant other and children. Hide the disease as much as possible from my employer.* [Male; 55-64 years; QC; AS, psoriasis]

^a^AB: Alberta.

^b^BC: British Columbia.

^c^RA: rheumatoid arthritis.

^d^QC: Quebec.

^e^AS: ankylosing spondylitis.

### Socioeconomic Vulnerabilities

Respondents explained how they simply did not have the energy to concurrently maintain employment and family responsibilities and attend to their own health, which resulted in financial strain (Table 3: F1, F2, and G1). They spoke of “falling through the cracks” between health care and social systems because eligibility requirements for programs denied them access. They described experiences where the health system or government priorities and budget constraints shifted definitions of disability in ways that excluded them from accessing the pensions or resources they needed or relied upon in the past (Table 3: G2 and G3). Some respondents reported difficulty in being taken seriously by their doctors (Table 3: K1 and K3) and consequently suffered setbacks in their treatment and health or expended time and effort coordinating and seeking out proper health care (Table 3: K2 and K4). For those living alone, their living arrangement was frequently cited as exacerbating the negative effects that their disease or diseases have on their quality of life (Table 3: F2 and K4).

Repeatedly, respondents explained how their disease makes them economically vulnerable because of employment insecurity or loss and the high cost of treatment and medication. It was difficult to buy items such as healthy food or services not funded by health plans to help them prevent complications (Table 3: H1, H2, and H3). The high cost of biologics as well as their unpredictable and potentially serious side effects or worries about long-term effectiveness were a burden common to many respondents regardless of diagnosis (Table 3: J1 and J2). The pressure and stress of dealing with health and social systems fostered a fear of the future and what it might hold (Table 3: J2).

**Table 3:** Socioeconomic vulnerabilities.

Subtheme	Quotes
Financial strain	F1: *Full-time employment was not possible so there were financial concerns because I could only work part-time. Reduced finances because of part-time employment, coping with the physical challenges of household tasks like cleaning, shopping, cooking, having enough energy to socialize after doing essentials, not feeling like a burden to my family.* [Female; 55-64 years; BC^a^; psoriasis, RA^b^, OA^c^]
	F2: *Chronic pain, immune symptoms, inflammation, quality of life. Financial burden of not working, being single and living alone in Vancouver. Having energy to eat well when I am flared up, and one of the biggest, is that patients really do need to be our own advocates in order to avoid falling through cracks in the system.* [Female; 35-44 years; BC; endometriosis, inflammatory bowel disease]
Disability programs are inadequate and restrictive	G1: *Having a chronic, painful illness at a younger age means trying to juggle the symptoms (including crippling fatigue) with children, spouse, home responsibilities, and work. Combined with a long commute, it's almost impossible for me to spend any time taking care of myself, like getting more sleep or exercise, or eating better. I want to get better, and I know I need to take better care of myself, but I can't figure out how to make it work. I don't think there are any programs or support (at least I’ve never heard of any) for young people managing these types of issues. Most programs are geared towards the elderly.* [Female; 35-44 years; BC; RA]
	G2: *Trying to manage working, running a house and exercising on limited energy. Helping my children deal with the unknown and day to day issues of a chronically ill mom and finally, disability programs do not deal well with chronically ill people able to work part time.* [Female; 45-54 years; AB^d^; lupus, Sjogren, polymyositis]
	G3: *How to earn a living. No money means no options. If the government keeps rejecting disability claims because chronic arthritis is not disabled enough how does one make money? Cannot go to swimming pool or buy decent quality food to help fight inflammation, cycle becomes a revolving door.* [Male; 35-44 years; BC; AS^e^]
Added costs of maintaining health with a chronic disease	H1: *[I feel the] financial burden of not having a lot of (realistic) job options - costs associated with disease management that have no coverage (i.e., are not prescription drugs), “high cost, high quality foods,” alternative health care, gym/pool memberships, equipment, living with chronic pain -not sleeping- not easy in social situations, (bathroom availability, food/water sources available).* [Female; 45-54 years; BC; UC^f^]
	H2: *The cost of helpful therapies e.g., massage, medications, etc. [I’m] now on a disability pension, the things that support my quality of life are difficult to get.* [Female; 55-64 years; BC; lupus]
	H3: *I cannot afford to eat a good diet, and pay for my medications, treatments, supplements, and pay my bills on what I get from disability and the small amount I am allowed to earn. I have to choose to eat well or take the medications, I can't afford both. Without both, I cannot manage my disease well.* [Female; 35-44 years; BC; lupus]
The cost of biologics	J1: *My biggest concern is that the [biologic] which currently controls my RA could someday become less effective or stop working altogether, and that no other treatment will be effective. My other concern is that perhaps despite the [biologic], I could still be slowly incurring joint damage leading someday to disability and deformity. The greatest burden is managing the high cost of biologics.* [Female; 55-64 years; ON^g^; RA]
	J2: *I’m fearful of what my future holds in terms of health problems. In addition, because of the symptoms I am unable to work or obtain employment and this impacts me severely financially. The medications I am on are also costly and again cause financial burden.* [Female; 45-54 years; BC; lupus]
Time and effort to manage own health care	K1: *My biggest concerns are: 1) Doctors listen to what I am saying about my symptoms and not rely solely on test outcomes. 2) Take seriously my description of being in severe pain.* [Female; 55-64 years; MB^h^; Crohn; OA]
	K2: *I have to be the captain (or co-captain) of my health care team. I have poor access to my medical records. There are a lot of out-of-pocket expenses. Important to be highly health literate, taking medications forever, social stigma, low level of socializing, hard to work full time, more disability in future.* [Female; 65-74 years; ON; RA, Sjogren]
	K3: *Although I appreciate my doctors, I often feel they do not appreciate me asking questions or taking part in my own care. I am very experienced with this disease. I find they push for invasive diagnostic procedures promising no pain, when in the end there is pain. I know they are trying to help but I think treating UC [ulcerative colitis] patients is not their favourite thing. I wish health professionals learned more about the day to day challenges of living with a disease such as colitis.* [Female; 55-64 years; BC; UC]
	K4: *Constant pain, not knowing when my hip will pop out of its socket, long term disability from work (12+ years ago) and dealing with CPP [pension plan] and long-term shortage of money due to being unable to work and do the activities I would like to do, poor balance and living alone in a small island place when my present doctor and specialists are in the greater Vancouver area. I am NOT allowed to have 2 G.P.s - so must keep the M.D. who has helped me with my condition(s) for the last 12+ years and take the ferry and bus to appointments in Victoria and Vancouver as Travel Assistance Plan forms can't be forwarded to me as the Medical Clinic on this island won't give me TAP forms and the G.P. and staff in New Westminster have no idea how to locate these forms - a nightmare!* [Female; 55-64 years; BC; arthritis]

^a^BC: British Columbia.

^b^RA: rheumatoid arthritis.

^c^OA: osteoarthritis

^d^AB: Alberta.

^e^AS: ankylosing spondylitis.

^f^UC: ulcerative colitis.

^g^ON: Ontario.

^h^MB: Manitoba.

### Staying Positive

Although the above 3 themes speak about undesirable consequences of inflammatory disease, there is a contrasting narrative arising from these written entries that tells a more positive story of resilience and adaptation. Examples of collaborative care, where health professionals and patients work together to ensure treatment both parties found appropriate, was 1 example. Some respondents shared strategies they found effective:

I have received strong encouragement from my nephrologist and my rheumatologists to exercise, and a wonderful, now-retired rheumatologist referred me to a physio. Physical activity has been absolutely critical to my well-being. I’m so grateful!

Female; age 45-54 years; BC; gout, lupus, Sjogren

Another respondent described collaborative care:

My team of doctors listen to me, respect me and believe me. We work together to get me healthier. They are proactive in searching for answers and options. My acupuncturist and massage therapist have the same attitudes as the doctors. The very supportive environment this creates helps me stay focused on getting healthier; even when the pain is so bad I feel like I can’t move, I know that it’s my job to get out and walk and exercise. They’re all doing what they can to help me and I, in turn, must do my part. Without their support, I’m sure there are many times I would have given up.

Female; 55-64 years; BC; psoriasis, rheumatoid arthritis

Staying positive is presented as a serious but necessary challenge:

Pain management is my biggest burden. You must be constantly aware of your physical limitations and not make them your focus. Forcing yourself to stay active and positive despite how you feel.

Female; 55-64 years; QC; ankylosing spondylitis

Keeping a positive attitude was cast by some participants as a struggle against fears and anxieties about the future. The following respondent shared reflections across several decades of living with inflammatory diseases, illustrating the episodic nature of both the disease and positive attitude:

Initially (in my 20s) with total body coverage of psoriasis, I was concerned I would never meet anyone that could tolerate how “ugly” I was (felt); then with onset of PsA [psoriatic arthritis] in my late 30’s my main concern was keeping mobile and being able to care for our daughter. Now in my 70’s after 10 years of clear skin and pain free joints due to the effectiveness of the [medication] injections, I’m concerned the drug is losing its effectiveness and I will lose the wonderful “normal” life I have had – walking, cycling, dancing, sleeping, and pain free (almost) during the night.

Female, BC, 65-74 years, psoriatic arthritis, Sjogren

## Discussion

### Principal Findings

Disruptions to daily life, systemic vulnerability, coping with (in)visible disability, and staying positive are interconnected aspects of living with chronic inflammatory diseases. Written passages from Canadians living with inflammatory joint, skin, or bowel diseases support 4 intertwined narratives, none of which exists in isolation, illustrating challenges encountered on a regular basis, regardless of diagnosis. The reasons for disruptions differed across diseases and individual experiences, but the overall consequences were quite similar. For example, the difficulty of maintaining steady employment and income threatens financial stability; consequently, one is less able to afford the goods and services that, alongside medical care, support a healthy lifestyle that makes the difference between inflammatory disease being a manageable condition rather than a miserable one. When daily life is disrupted, the relationships that hold peoples’ lives together begin to unravel, whether it is a relationship with one’s employer who sees inflammatory disease as a liability or one’s coworkers, friends, or family who do not understand the burdens imposed by the disease. Many respondents stated a need to try to hide their disability, having encountered or anticipated a lack of understanding or compassion from those around them as essential to supporting a positive self-identity. Managing diseases, relationships, and life roles was a balancing act, consistent with prior smaller but more in-depth studies [12,14,15]. Thus, this survey of a large number of patients confirms the experiences described in prior research.

Some respondents regarded the responsibility to maintain a positive attitude while coping with chronic pain and disability as an ongoing mental and emotional challenge. However, this was not a universal experience because other respondents appeared to have mastered a positive perspective. They dismissed disease-related challenges as part of life and focused on things that mattered to them, such as enjoying with family and friends and enjoying activities, regardless of their health conditions. As the survey item used the phrase “burdens and responsibilities,” it solicited responses regarding difficult experiences; however, the small number of respondents who spontaneously presented a positive narrative instead was nonetheless critical. What is unknown, given the limitations of a single, written submission from each participant, is the extent to which a positive perspective can be sustained by the individual’s resources such as access to health care, economic security, the presence of strong social networks, or responsibilities like caring for others—all of which contribute to health disparities. It is also possible that these descriptions of resilience, such as inflammatory diseases, are episodic or reflect a stage of adaptation to living with a long-term condition [26]. On the basis of the findings of this study, those with highly positive descriptions credited respectful, collaborative relationships with health care providers and understanding family, friends, and employers with supporting their outlook on life.

The findings suggest that many respondents’ needs are not well served by a system that isolates each individual problem to the exclusion of seeing the bigger picture. This bolsters evidence for a biopsychosocial approach that integrates the social experiences of patients with the psychological and physical impacts of their disease or diseases. Finding solutions to the consequences of long-term illness requires a patient-led research agenda because as Rose argues, public and patient engagements are forms of civic participation and citizenship that work toward the democratization of science [27]. Patient engagement in research is an avenue for their concerns and priorities to be represented, and by extension, better addressed in health and social sectors. This confirmatory study with 540 participants shows that many health needs are unmet from the patient perspective, explained in part by lack of attention to social determinants of health. That patients seek symptom relief, strategies to support daily life, a functioning social safety net, and empathic social support and health services is not new, but the repetition across multiple patient experiences indicates these important and long-standing issues have yet to be resolved. This suggests one role for these findings is to inform system, policy, and service delivery change needed to resolve these issues.

Examples for engaging patients in research are widely available [28-30], and our experience had both strengths and room for improvement. Researchers are generally motivated to try public engagement because they feel it will increase the relevance of their findings, whereas patients may be motivated by the desire for more user-oriented services [28,18]. A moral rationale for patient-partnered research is that it honors and respects the patients’ voice, supports participation, minimizes occupational disruption, and advances a role for patient organizations in public education of the need for societal supports, large and small [29]. Moving forward, a measure of patient engagement in research that can serve as a guide for assessing the quality and depth of patient engagement in a given project may be useful and lead to more user-oriented research [30-32].

### Strengths and Limitations

The large sample in this study was a major strength as it ensured that all relevant topics to the study populations were uncovered. There are 2 key limitations. First, the survey was originally designed to inform research priorities and questions and not as an original research study; thus, items were neither standardized nor pilot tested. Second, the single open-ended question is a minimalist form of data elicitation, and although this paper presents a qualitative analysis, it was not a prospectively designed qualitative study. Although the opportunity to probe further (as in other forms of qualitative inquiry) was not possible with this mode of collecting written narratives, we had narrative texts from over 500 Canadians. Typical qualitative research involves theoretically informed designs with in-depth descriptions from a small number of participants. What was lost in depth is counterbalanced by breadth, enhancing transferability to Canadians with similar diagnoses. We believe that this study is a valuable contribution to inflammatory disease research, despite the methodological limitations of qualitative analysis of open-ended survey questions. Through its rigorous self-awareness of the limitations of its data, relevance in identifying cross-cutting issues from other studies, and engagement with patient partners, this study meets the criteria set out by LaDonna et al that mark it as an exception to the general weakness of such methodological designs [33].

Prior studies of living with chronic inflammatory diseases have eloquently illustrated the burden and responsibility within a disease group such as arthritis, inflammatory bowel disease, or psoriasis [7,14,17]; our survey extends those findings across a large number of people and inflammatory diseases. The survey format allowed respondents anonymity and freedom to speak their thoughts, in contrast to the more personal interaction of a research interview. An advantage of this approach may be a lesser likelihood of social desirability–shaping responses, that is, that respondents tell the researcher what they believe the researcher wants or expects to hear. The limitation, however, of having to take responses at face value without more probing means that some clarity of meaning may be lost. As a survey administered “by patients for patients,” a platform was provided for critical input from respondents that may otherwise be elusive in more structured quantitative and qualitative studies alike.

Public engagement in research happens most often at the stage when researchers need patient input to help identify a relevant research question [34]. Although this was the case with our study, patient partners remained engaged throughout the research process, beyond the initial phase when it is advantageous to securing funding. We consider it a strength of this analysis that it was undertaken with respect to the values of patient and public engagement outlined by Gradinger et al, namely, a concern for the ethical, political, and normative values as well as for the process-based values such as respect, partnership, and equality [35]. When initiated, our survey was intended to demonstrate to the funding agency that patients were actively engaged in the proposal from inception. However, the insight gained from the survey not only helped develop a proposal to better understand the medical complications of inflammatory diseases but it also generated substantial data on the social and emotional consequences that are integrally tied to the provision of health care services and the patient-provider relationship.

### Conclusions

Analysis of written responses to a survey created by patients for patients living with chronic inflammatory diseases shows many common experiences regardless of diagnosis, including disruptions to daily life and socioeconomic vulnerabilities that create and contribute to worries about the future. The issues raised by this paper concern the interrelatedness of health, social, and economic support systems and the gaps between them that create barriers to finding and accessing the services people with inflammatory diseases need to maintain their health. However, respondents also describe examples of patient-provider partnerships and social systems that contribute to personal resilience and capacity to participate in life. This paper brings together the narratives of a large sample of patients to emphasize commonalities in the experiences of inflammatory disease patients, who are often analyzed in the isolation of their specific diseases than as a broad category. It illustrates a meaningful collaboration between patients and researchers that suggests a patient-led research agenda in chronic inflammatory diseases would foreground the role of the social determinants of health in shaping disease outcomes. Such findings should inform policy and service delivery through system change.

## Abbreviations

AB: Alberta
AS: ankylosing spondylitis
BC: British Columbia
MB: Manitoba
ON: Ontario
OA: osteoarthritis
PRECISION: PREventing Complications from Inflammatory Skin, joint, and bowel conditions
QC: Quebec
RA: rheumatoid arthritis
SK: Saskatchewan
UC: ulcerative colitis

## References

[1] Domecq JP, Prutsky G, Elraiyah T, Wang Z, Nabhan M, Shippee N, Brito JP, Boehmer K, Hasan R, Firwana B, Erwin P, Eton D, Sloan J, Montori V, Asi N, Dabrh AM, Murad MH (2014). Patient engagement in research: a systematic review. BMC Health Serv Res.

[2] de Wit MP, Elberse JE, Broerse JE, Abma TA (2015). Do not forget the professional--the value of the FIRST model for guiding the structural involvement of patients in rheumatology research. Health Expect.

[3] Smolen JS, Aletaha D, McInnes IB (2016). Rheumatoid arthritis. Lancet.

[4] Boehncke W, Schön MP (2015). Psoriasis. Lancet.

[5] Torres J, Mehandru S, Colombel J, Peyrin-Biroulet L (2017). Crohn's disease. Lancet.

[6] Becker HM, Grigat D, Ghosh S, Kaplan GG, Dieleman L, Wine E, Fedorak RN, Fernandes A, Panaccione R, Barkema HW (2015). Living with inflammatory bowel disease: a Crohn's and Colitis Canada survey. Can J Gastroenterol Hepatol.

[7] Katz PP, Morris A, Yelin E (2006). Prevalence and predictors of disability in valued life activities among individuals with rheumatoid arthritis. Ann Rheum Dis.

[8] Lynde CW, Poulin Y, Guenther L, Jackson C (2009). The burden of psoriasis in Canada: insights from the pSoriasis Knowledge IN Canada (SKIN) survey. J Cutan Med Surg.

[9] Meyer N, Paul C, Feneron D, Bardoulat I, Thiriet C, Camara C, Sid-Mohand D, Le Pen C, Ortonne JP (2010). Psoriasis: an epidemiological evaluation of disease burden in 590 patients. J Eur Acad Dermatol Venereol.

[10] (2011). Arthritis Alliance.

[11] Zhang W, Anis AH (2011). The economic burden of rheumatoid arthritis: beyond health care costs. Clin Rheumatol.

[12] McDonald HN, Dietrich T, Townsend A, Li LC, Cox S, Backman CL (2012). Exploring occupational disruption among women after onset of rheumatoid arthritis. Arthritis Care Res (Hoboken).

[13] Baker CS, Foley PA, Braue A (2013). Psoriasis uncovered--measuring burden of disease impact in a survey of Australians with psoriasis. Australas J Dermatol.

[14] Hall NJ, Rubin G, Dougall A, Hungin APS, Neely J (2005). The fight for 'health-related normality': a qualitative study of the experiences of individuals living with established inflammatory bowel disease (ibd). J Health Psychol.

[15] Sykes DN, Fletcher P, Schneider M (2015). Balancing my disease: women's perspectives of living with inflammatory bowel disease. J Clin Nurs.

[16] Kimball AB, Jacobson C, Weiss S, Vreeland MG, Wu Y (2005). The psychosocial burden of psoriasis. Am J Clin Dermatol.

[17] Leino M, Mustonen A, Mattila K, Koulu L, Tuominen R (2014). Perceived impact of psoriasis on leisure-time activities. Eur J Dermatol.

[18] Hewlett S, de Wit M, Richards P, Quest E, Hughes R, Heiberg T, Kirwan J (2006). Patients and professionals as research partners: challenges, practicalities, and benefits. Arthritis Rheum.

[19] Price A, Schroter S, Snow R, Hicks M, Harmston R, Staniszewska S, Parker S, Richards T (2018). Frequency of reporting on patient and public involvement (PPI) in research studies published in a general medical journal: a descriptive study. BMJ Open.

[20] (2015). Arthritis Research Canada.

[21] Koehn C, Attara G, Stordy A, Montie P, PRECISION (2014). The University of British Columbia.

[22] Hannah DR, Lautsch BA (2010). Counting in qualitative research: why to conduct it, when to avoid it, and when to closet it. JMI.

[23] Reissman CK (2004). Sage Encyclopedia of Social Science Research Methods.

[24] Hsieh HF, Shannon S (2005). Three approaches to qualitative content analysis. Qual Health Res.

[25] Braun V, Clarke V (2006). Using thematic analysis in psychology. Qual Res Psychol.

[26] Aujoulat I, Marcolongo R, Bonadiman L, Deccache A (2008). Reconsidering patient empowerment in chronic illness: a critique of models of self-efficacy and bodily control. Soc Sci Med.

[27] Rose D (2014). Patient and public involvement in health research: ethical imperative and/or radical challenge?. J Health Psychol.

[28] Staniszewska S, Jones N, Newburn M, Marshall S (2007). User involvement in the development of a research bid: barriers, enablers and impacts. Health Expect.

[29] Prior SJ, Campbell S (2018). Patient and family involvement: a discussion of co-led redesign of healthcare services. J Participat Med.

[30] Shen S, Doyle-Thomas KA, Beesley L, Karmali A, Williams L, Tanel N, McPherson AC (2017). How and why should we engage parents as co-researchers in health research? A scoping review of current practices. Health Expect.

[31] Brett JO, Staniszewska S, Mockford C, Herron-Marx S, Hughes JE, Tysall C, Suleman R (2014). Mapping the impact of patient and public involvement on health and social care research: a systematic review. Health Expect.

[32] Hamilton CB, Hoens A, Backman C, McKinnon A, McQuitty S, English K, Li L (2018). An empirically based conceptual framework for fostering meaningful patient engagement in research. Health Expect.

[33] LaDonna KA, Taylor T, Lingard L (2018). Why open-ended survey questions are unlikely to support rigorous qualitative insights. Acad Med.

[34] Boote J, Wong R, Booth A (2015). 'Talking the talk or walking the walk?' A bibliometric review of the literature on public involvement in health research published between 1995 and 2009. Health Expect.

[35] Gradinger F, Britten N, Wyatt K, Froggatt K, Gibson A, Jacoby A, Lobban F, Mayes D, Snape D, Rawcliffe T, Popay J (2015). Values associated with public involvement in health and social care research: a narrative review. Health Expect.

